# **β**-Catenin Does Not Confer Tumorigenicity When Introduced into Partially Transformed Human Mesenchymal Stem Cells

**DOI:** 10.1155/2012/164803

**Published:** 2012-10-18

**Authors:** Sajida Piperdi, Lukas Austin-Page, David Geller, Manpreet Ahluwalia, Sarah Gorlick, Jonathan Gill, Amy Park, Wendong Zhang, Nan Li, So Hak Chung, Richard Gorlick

**Affiliations:** ^1^The Department of Pediatrics, Albert Einstein College of Medicine, Yeshiva University, Bronx, NY 10461, USA; ^2^The Department of Pediatrics, Children's Hospital Los Angeles, Los Angeles, CA 90027, USA; ^3^The Department of Orthopaedic Surgery, Montefiore Medical Center, Bronx, NY 10467, USA; ^4^Division Hematology/Oncology, Department of Pediatrics, The Children's Hospital at Montefiore, Room 300, Rosenthal Building, 3415 Bainbridge Avenue, Bronx, NY 10467, USA; ^5^Oncology Section, The Department of Orthopedics Surgery, First Affiliated Hospital of PLA General Hospital, Beijing 100037, China; ^6^The Department of Orthopedics Surgery, College of Medicine, Kosin University Gospel Hospital, Busan 602-702, Republic of Korea; ^7^Department of Pediatrics and Molecular Pharmacology, Albert Einstein College of Medicine, Yeshiva University, Bronx, NY 10461, USA; ^8^Division of Hematology/Oncology, Department of Pediatrics, The Children's Hospital at Montefiore, Room 300, Rosenthal Building, 3415 Bainbridge Avenue, Bronx, NY 10467, USA

## Abstract

Although osteosarcoma is the most common primary malignant bone tumor in children and adolescents, its cell of origin and the genetic alterations are unclear. Previous studies have shown that serially introducing hTERT, SV40 large TAg, and H-Ras transforms human mesenchymal stem cells into two distinct sarcomas cell populations, but they do not form osteoid. In this study, **β**-catenin was introduced into mesenchymal stem cells already containing hTERT and SV40 large TAg to analyze if this resulted in a model which more closely recapitulated osteosarcoma. *Results*. Regardless of the level of induced **β**-catenin expression in the stable transfectants, there were no marked differences induced in their phenotype or invasion and migration capacity. Perhaps more importantly, none of them formed tumors when injected into immunocompromised mice. Moreover, the resulting transformed cells could be induced to osteogenic and chondrogenic differentiation but not to adipogenic differentiation. *Conclusions*. **β**-catenin, although fostering osteogenic differentiation, does not induce the malignant features and tumorigenicity conveyed by oncogenic H-RAS when introduced into partly transformed mesenchymal stem cells. This may have implications for the role of **β**-catenin in osteosarcoma pathogenesis. It also may suggest that adipogenesis is an earlier branch point than osteogenesis and chondrogenesis in normal mesenchymal differentiation.

## 1. Introduction

Osteosarcoma is the most common primary malignant bone tumor in children and young adults [1, 2]. The lack of a precursor lesion combined with the genetic complexity of osteosarcoma has limited understanding the etiology of this disease. Microscopically, osteosarcoma is defined as a malignant spindle cell tumor that produces osteoid. The presence of this bony matrix has led to the traditional viewpoint that the tumor is derived from the osteoblast. However, depending on the type of the predominant matrix, histological subtypes of osteosarcoma including chondroblastic, fibroblastic, and osteoblastic subtypes are defined [3, 4]. The existence of these histologic subtypes suggests the tumors have a multilineage differentiation capacity and suggest that the cell of origin is more pluripotent than an osteoblast [5]. To date, the factors associated with an osteosarcoma having a particular histological appearance are poorly understood. Osteosarcoma could arise from a cell anywhere from a mesenchymal stem cell (MSC) to an osteoblast and originate from various cellular pools existing in the bone marrow, the growth plates, or the periosteum [6]. Identifying the cell of origin and the molecular basis of osteosarcoma may be of critical clinical importance [7].

The extremely complex Wnt pathway encodes highly conserved genes and secreted proteins, which modulate cell fate and cell proliferation during embryonic development and carcinogenesis through activation of receptor-mediated signaling pathways [8–10]. In the most well-known and highly conserved canonical Wnt pathway, the presence of Wnt triggers the cascade of receptor activation causing the inactivation of the intracellular enzyme GSK3 and the tumor suppressor APC, key factors that promote the degradation of cytoplasmic pool of *β*-catenin, the key downstream mediator of Wnt-signaling pathway, allowing the translocation of *β*-catenin to the nucleus resulting in transcriptional activation of downstream targets, many of which are involved in embryonic development and oncogenesis. The activation of Wnt pathway and *β*-catenin has been implicated in the pathogenesis and progression of an increasingly number of human malignancies, including colorectal cancer, melanoma, myeloma, and lung cancer [11–13]. It has been reported that in osteosarcoma, there are overexpressions of numerous Wnt components [14, 15] as well as the epigenetic silencing of Wnt inhibitory factor 1 and frizzled related protein 3 [16, 17]. In addition, *β*-catenin mutation and elevated levels of nuclear *β*-catenin have been also noted in osteosarcoma and associated with lung metastasis [18–21], highlighting the potential association between the Wnt-*β*-catenin signaling in the development and progression of osteosarcoma.

In order to better understand osteosarcomas genetic complexity, our efforts have been directed towards developing a tumor which recapitulates osteosarcomas phenotype by introducing defined genetic elements into human mesenchymal stem cells (hMSCs). Initially, hMSCs were transformed by the serial introduction of hTERT, SV40 TAg and H-Ras as had been described previously for transformation of other normal cell types [22, 23]. The resulting cells were oncogenic, capable of producing tumors in mice, but histologically they were a malignant spindle cell tumor which did not produce osteoid, hence they were not osteosarcoma [23]. It was hypothesized that introducing a genetic alteration inducing osteogenic differentiation may result in the desired phenotype. Based on the aforementioned involvement of *β*-catenin in both tumor development and osteogenic differentiation, *β*-catenin was introduced into hMSC already transformed by hTERT and SV40 TAg. In this paper, the creation of these cells along with their characterization is reported, providing insights into the potential role of *β*-catenin in osteosarcoma pathogenesis.

## 2. Materials and Methods

### 2.1. Cell Culture

hMSCs and their transformed derivatives, transfected with hTERT and SV40 TAg, named, MSC-TS, were obtained, produced, and cultured as previously described [23] in MSC medium (Lonza, Walkersville, MD, USA) at 37′C with 5% CO_2_. Cell morphologies were observed and pictures were taken using a Nikon Inverted Microscope ECLIPSE TE200 attached to a cooled charge coupled device (Diagnostic Instruments, Sterling Heights, MI, USA). HT1080, HOS, and NIH 3T3 cells were purchased from American Tissue Type Culture Collection (Manassas, VA, USA) and cultured as per their instructions.

### 2.2. Viral Transfection, Creating Stably Transfected Cell Lines and Immunoblotting

A full length cDNA clone of *β*-catenin constructed in pCMV6-XL5 vector was purchased from OriGene (Rockville, MD, USA). The lentiviral plasmid pLenti-*β*-catenin-blast was constructed by subcloning the above *β*-catenin entry clone using pLenti6.2/V5-DEST Gateway Vector Kit and ViralPower Lentiviral Expression Systems (Invitrogen, Carlsbad, CA, USA). PLenti-*β*-catenin-blast was transiently transfected into the 293FT packaging cell line (Invitrogen, CA, USA). Viral stock was harvested at 30 hours and MSC-TS cells were infected with 6 *μ*g/mL polybrene when they were at 50% confluence. Following 24 hours of coculture, drug selection of infected cells was performed using 3 *μ*g per mL blasticidin. Ten drug resistant colonies were picked up after 6 weeks and were separately subcultured. Expression of *β*-catenin was measured by immunoblotting 15 *μ*g total protein with *β*-catenin monoclonal antibody, 6B3 (Cell Signaling Technology, Beverly, MA, USA), according to the manufacturer's instructions. Clones which had the highest, lowest, and intermediate *β*-catenin level were selected for further analysis.

### 2.3. Immunocytochemical Analysis

Immunocytochemical staining of *β*-catenin in cultured cells was carried out using immunofluorescence staining methods. Cells were plated and cultured to 30–40% confluence on four-well chamber slides. Cells were then washed with PBS, fixed in ice-cold methanol for 5 min, washed, permeabilized with 0.25% Triton X-100 in PBS, and washed again. Nonspecific sites were blocked with 10% normal serum in 1% BSA in PBS for 1 h at RT. Cells were then incubated with 2 ug/mL rabbit polyclonal anti-*β*-catenin antibody (Millipore, Billerica, MA, USA) or purified IgG for isotype controls overnight at 4′. The next day, cells were incubated with a 1 : 250 dilution of goat anti-rabbit IgG secondary antibody conjugated to Alexa-488 green fluorescence (Life Technologies, Grand Island, NY, USA) for 1 h at RT. Cells were then washed and counterstained with prolong gold antifade reagent with DAPI (Life Technologies, Grand Island, NY). Immunostaining was visualized by Zeiss Axio Observer inverted microscope at 63X magnification.

### 2.4. Subcutaneous and Orthotopic Tumorigenicity Assays

Six-to-eight-week-old CB-17 SCID mice (Taconic, Germantown, NY, USA) were injected with the MSC-TS and now also transfected with *β*-catenin, which were named MSC-TSB, as described previously [23]. Parallel subcutaneous injections of MSC-TS and a subline of MSC-TS transfected with activated H-Ras and previously named MSC-TSR6 were performed as negative and positive controls, respectively. Twelve mice were used for each cell line, regularly checked for tumor formation, and all experiments were performed in accordance with protocols approved by the Institutional Animal Care and Use Committee of the Albert Einstein College of Medicine.

### 2.5. Motility/Migration and Invasion Assays

Motility (random migration) was measured by the wound healing assay as previously described [23]. Cells were cultured in serum-free media overnight before creating wounds. Photos were taken every 12 hours until 72 hours at the same region. The width of the scratch wounds was measured in Image J Software, and relative change in scratch wound width was calculated for 0 and 12 hr. Migration (haptotaxis) was measured using the Chemicon QCM Quantitative Cell Migration Assay (Millipore, Billerica, MA, USA). Again, cells were serum-starved overnight before being seeded into Boyden chambers. Cells that migrated to the outside of the chamber were stained and extracted in 300 *μ*L of extraction buffer. Absorbance at 562 nm was measured using a microplate reader (Bio-Rad, Hercules, CA, USA).

Invasion was measured using the Chemicon Cell Invasion Assay (Millipore, Billerica, MA, USA). Serum-starved cells were plated in the invasion chambers which have 8 *μ*m pore size polycarbonate membrane, over which a thin layer of extracellular matrix (ECM) is applied. The invasive cells which migrate through the matrix layer become attached to the bottom of the polycarbonate membrane, which is then stained and extracted.

### 2.6. Differentiation Assays

The osteogenic, adipogenic and chondrogenic differentiation capacity were measured according to manufacturer's instructions using an MSC osteogenesis kit, mesenchymal adipogenesis kit, and chondrogenic differentiation media with transforming growth factor (TGF-*β*3) (Lonza, Walkersville, MD, USA), respectively. Cells were cultured in differentiation induction medium for 4–8 weeks. Differentiated cells were stained with Alizarin Red, Oil Red O, and immunohistochemical staining of type II collagen using antibody collagen type II 003-02 (Santa Cruz, Santa Cruz, CA, USA), which can stain calcium, fat, and type II collagen, respectively, to verify formation of osteocytes, adipocytes, and chondrocytes, respectively. Chondrogenesis was also performed using a StemPro chondrogenesis differentiation kit (Invitrogen, Carlsbad, CA, USA) and was subsequently stained with alcian blue (Sigma-Aldrich, St. Louis, MO, USA) for proteoglycans produced by chondrocytes. Pictures were taken using a Nikon Inverted Microscope ECLIPSE TE200 attached to a CCD (Diagnostic Instruments, Sterling Heights, MI, USA).

### 2.7. Statistical Analysis

Student's *t* test was used to compare the difference between means. *P* < 0.05 was considered statistically significant. All of the data were analyzed using a statistical software package (SPSS 20.0, Chicago, IL, USA).

## 3. Results

### 3.1. Generation of Stably Transformed Human Mesenchymal Stem Cell Lines

**Figure 1:**
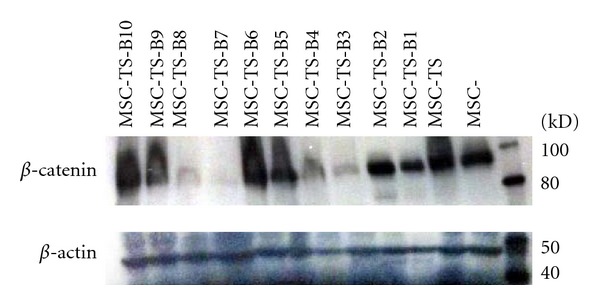
*β*-catenin protein expression was confirmed by western blot, with *β*-actin serving as a loading control. Lane 1: hMSC. Lane 2: hMSC-TS. Lane 3–12: hMSC-TSB clones 1 through 10.

Stably transformed MSC-TS cell lines had been developed previously using neomycin and puromycin as the selectable markers [23]. *β*-catenin was introduced into the MSC-TS cells using blasticidin as the selectable marker. This new stably transfected MSC derived line was named MSC-TSB with T representing hTERT, S representing SV40 Tag, and B representing *β*-catenin, respectively. The levels of *β*-catenin expression were confirmed by western blot (Figure 1). *β*-catenin was expressed at varying levels in the ten clones that were selected after transfection, and also to some extent, in the parental cell lines, MSC, and MSC-TS. Clones that had the highest level of *β*-catenin, MSC-TSB-H1 and MSC-TSB-H2, lowest or null *β*-catenin level, MSC-TSB-L3 and MSC-TSB-L7, and intermediate level, MSC-TSB-I9 and MSC-TSB-I10, were selected for further analysis of the impact of *β*-catenin on their behavior.

**Figure 2:**
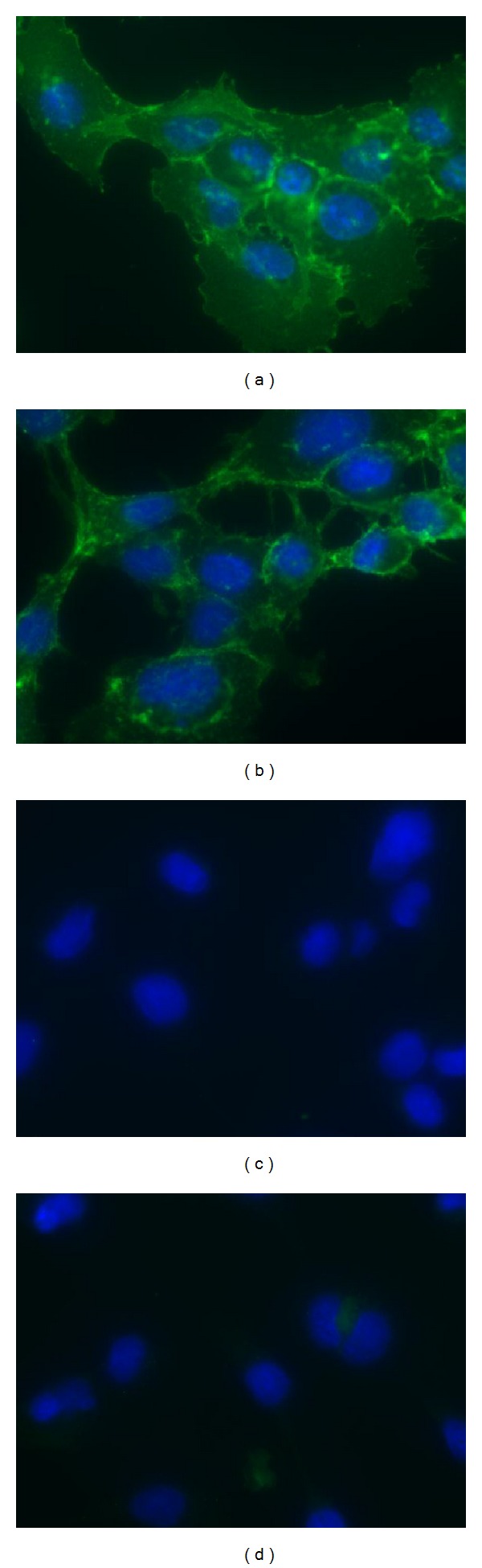
Representative pictures of immunofluorescence staining of *β*-catenin in tramsformed MSC-TSB cell lines. ((a)-(b)) *β*-catenin signal (green) was detected in both the cytoplasm and nucleus. ((c)-(d)) Their isotype control counterparts, respectively (63x original magnification).

No distinguishable changes in cellular localization of *β*-catenin (Figure 2), morphology, growth rate, and growth pattern were observed between these different colonies. The proliferation rate was markedly increased after *β*-catenin transfection, compared to MSC-TS with a population doubling time of less than 24 hours. Consistent with the behavior of the MSC-TSR cell line, the MSC-TSB cell lines also showed immortalization with the cells proliferating beyond 50 passages in culture.

### 3.2. Motility and Invasion

**Figure 3:**
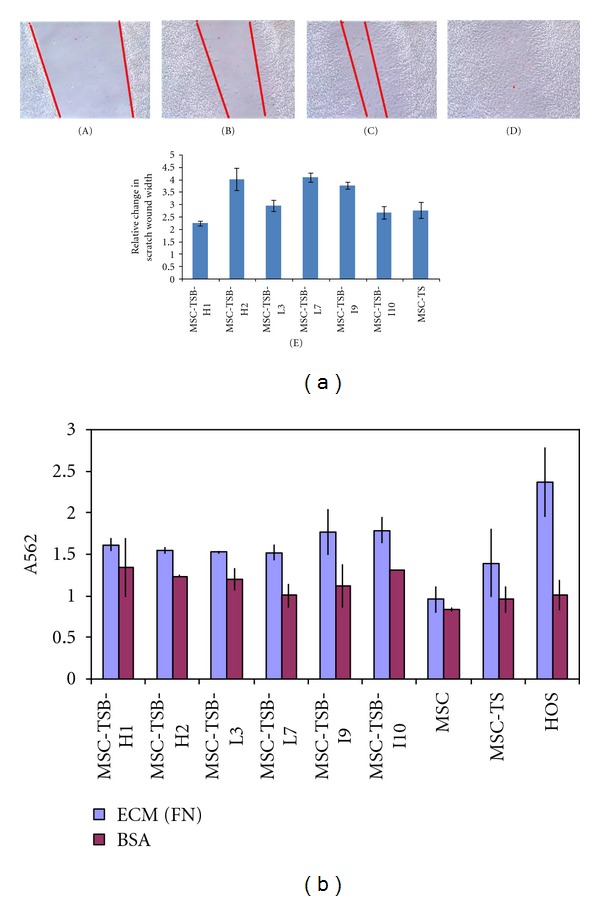
Migration assay. (a) A single representative wound healing assay (10x magnification), which showed no significant differences, *P* > 0.05, between the variants and the parental cell line MSC-TS at (A) 0 hour, (B) 12 hours, (C) 24 hours, and (D) 48 hours. (E) Quantitative measurement of wound gap by Image J software also showed no statistical significance between clones and the parental line. (b) A summary of the Boyden chamber experiment results; BSA coated wells serve as control along with HOS cells. Bars, standard deviation (SD). Experiments were replicated three times.

Two different kinds of motility were measured: random migration by wound healing assay (Figure 3(a)) and haptotaxis, a cell movement towards an immobilized ECM protein gradient as measured in the Boyden chamber system (Figure 3(b)). In these experiments, there were no major differences among the clones with differing *β*-catenin levels and the parental MSC-TS with *P* values > 0.05, statistically insignificant.

**Figure 4:**
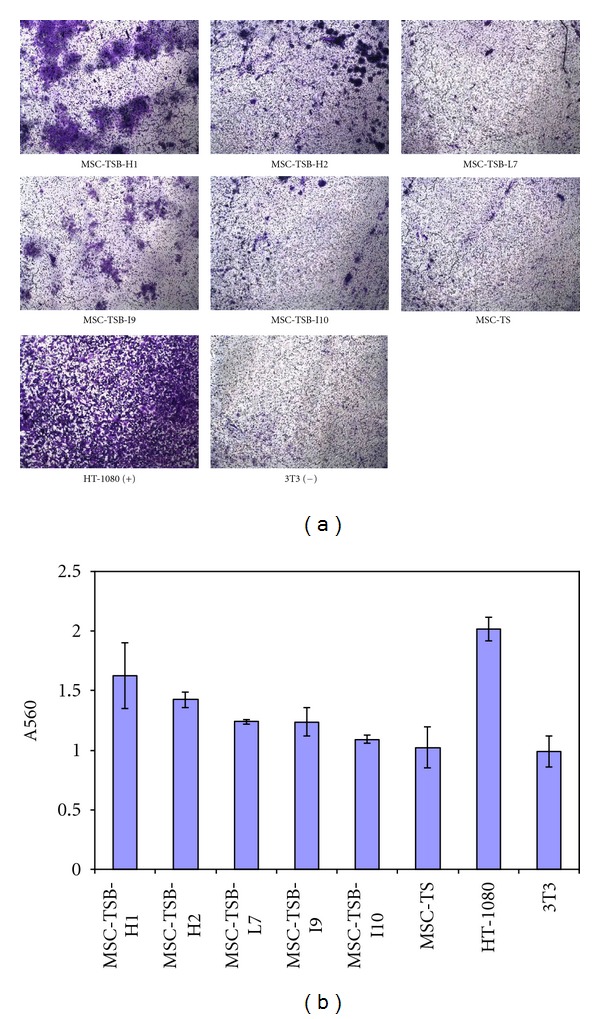
Cell invasion assay. (a) Pictures taken under microscope after staining (10x magnifications) to visualize the invasive cells across the polycarbonate membrane. (b) A summary of the invasion assay results after staining and extracting the cells outside the membrane. The highly invasive HT-1080 cell lines served as a positive control, and the noninvasive NIH-3T3 (3T3) cells served as a negative control. There is no statistically significant difference between the transformed clones with different *β*-catenin level and the parental MSC-TS, all *P* values are greater than 0.05.

Upon measuring the cellular invasiveness into the ECM, again, MSC-TSB clones did not show much variability between clones with differing *β*-catenin levels with the degree of invasion similar to that of MSC-TS, *P* values > 0.05. Their invasion capacity was far less than that of HT1080 which was used as the positive control. The noninvasive cell line 3T3 was used as the negative control (Figure 4). MSC-TSB-L3 was excluded due to a technical error.

### 3.3. Formation of Tumors in CB-17 SCID Mice

In tumorigenicity assays, no palpable tumor was found with any of the MSC-TSB clones in both subcutaneous and orthotopic-injected mice 6 weeks after the implantation. As a positive control, we used MSC-TSR6 which formed tumors in 7 out of 8 mice subcutaneously, and 4 out of 4 orthotopically. The parental MSC-TS also did not form tumors.

### 3.4. Changes in Multilineage Differentiation Capacity of Transformed hMSCs

**Figure 5:**
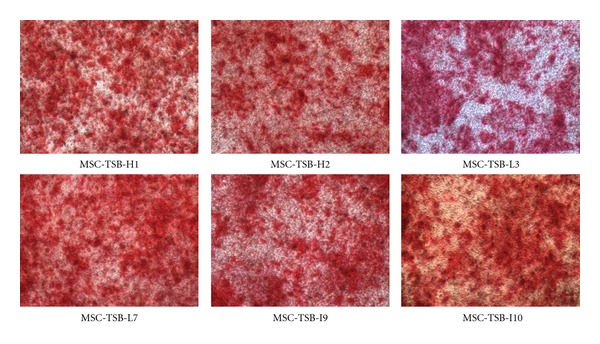
Osteogenic differentiation as demonstrated by staining with Alizarin Red 21 days after induction, 10x magnifications.

**Figure 6:**
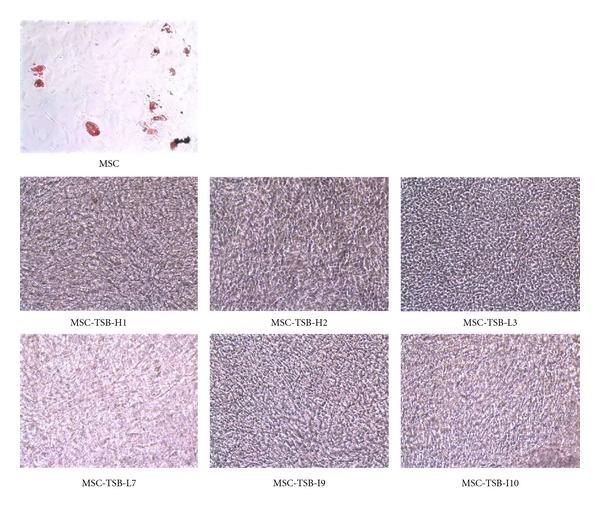
Adipogenic differentiation is not present as determined by staining with Oil Red O in the various MSC-TSB clones after 8 weeks in differentiation media. hMSC demonstrates adipogenic differentiation by Oil Red O staining after 4 weeks in differentiation media as a positive control (10x magnification).

**Figure 7:**
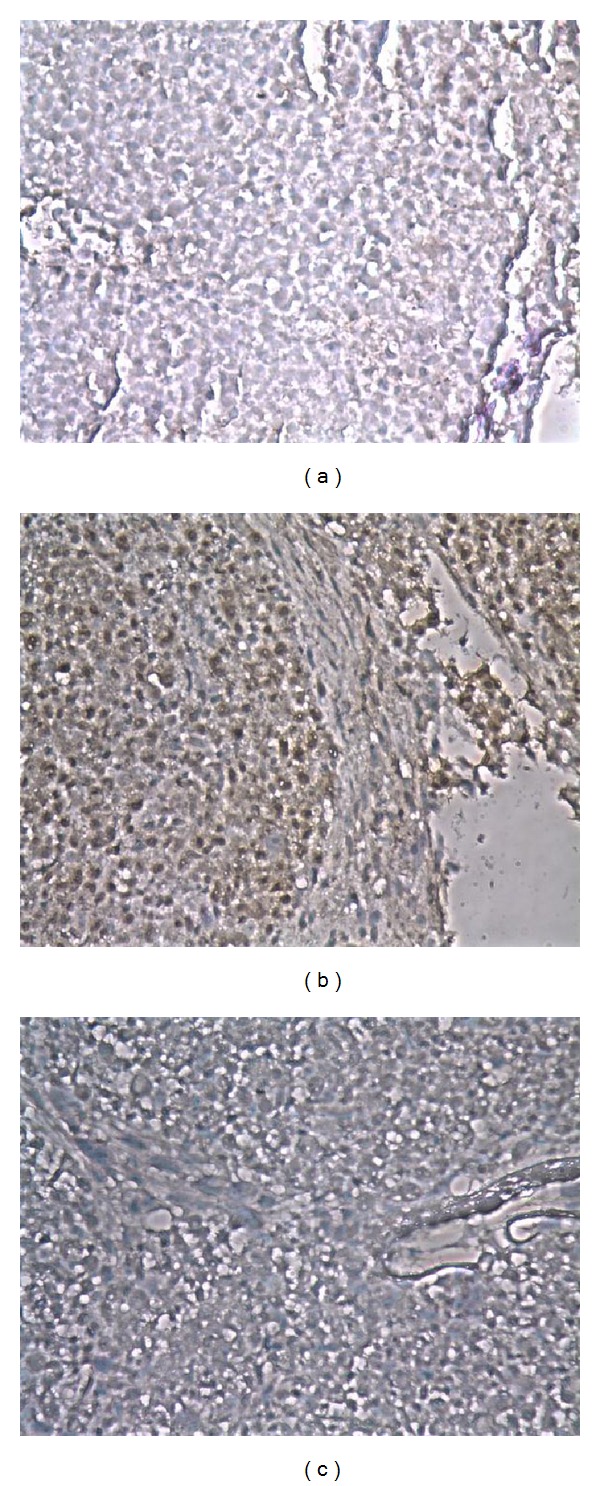
Representative pictures of staining of collagen type II after induction in chondrogenic differentiation media for 8 weeks. (a) Weakly positive, (b) positive for collagen, and (c) isotype control (10x magnification).

Osteogenic differentiation capacity was more rapid in MSC-TSB cell lines, once again independent of *β*-catenin expression level, as compared to MSC-TS. Strong intensity staining with Alizarin Red could be detected as early as 3 weeks in differentiation media (Figure 5). With the introduction of *β*-catenin, the cells lost the capacity for adipogenic differentiation as demonstrated by the lack of Oil Red O staining after being in differentiation media 8 weeks (Figure 6). For the chondrogenic differentiation assay, some MSC-TSB clones were found to stain weakly for collagen type II after 8 weeks of culture in chondrogenic induction media (Figure 7), whereas they were negative for proteoglycan by Alcian Blue staining (data not shown).

## 4. Discussion

In summary, *β*-catenin was successfully introduced into MSC-TS cells with the resulting cells being proliferative and immortalized, but without marked changes in motility or invasion and unable to produce orthotopic or heterotopic tumors in immunodeficient mice. Additionally these cells more rapidly underwent osteogenic differentiation but were unable to undergo adipogenic differentiation. Although these experiments did not produce an experimental model of osteosarcoma, some insights into the role of *β*-catenin in mesenchymal stem cell differentiation may be inferred. In addition, some implications may exist for intermediate stages of MSC differentiation.

There have been several reports indicating that murine mesenchymal stem cells could be transformed into malignant phenotype due to spontaneous mutations after many passages in culture, and therefore MSC could be a potential cell of origin of many sarcomas [24–26]. In addition, our previous study has been reported that the malignant transformation of hMSCs by serially introducing hTERT, SV40 Tag, and H-Ras produces a high-grade spindle cell sarcoma [23]. This led the current study to determine if an osteosarcoma phenotype could be produced by the introduction of *β*-catenin instead of H-Ras into the MSC-TS cells. Some of the resulting clones showed lower level of *β*-catenin than the parental MSC-TS on the western blot. In this context, it is unclear why these clones have lower protein levels; however, possible explanations include either posttranslational modification or negative feedback regulation of *β*-catenin/Tcf pathway in which increased *β*-catenin signalings induce Tcf-dependent *β*TrCP protein expression resulting in a rapid degradation of wild-type *β*-catenin [27]. The presence and subsequent use of multiple clones makes random events such as insertional mutagenesis less likely. As the resulting cells did not produce tumors in mice, these efforts were not successful in producing an osteosarcoma model system. Creation of a model system was intended to address questions of the cell of origin as well as the critical genetic steps in osteosarcoma development. In the context of these experiments, it is not possible to address these questions, as the failure to produce osteosarcoma could reflect once again an inappropriate use of hMSC as the starting point or an inappropriate selection of a gene for cellular transformation.

The rationale for the use of *β*-catenin to transform these cells has been discussed previously but the involvement of the Wnt pathway in osteosarcoma pathogenesis and progression is somewhat controversial [28]. Numerous studies have demonstrated an involvement of the Wnt pathway, including thorough expression of LRP5 as involved in osteosarcoma disease progression [16, 18, 19, 29, 30]. More specifically, previous studies have shown that downregulating the Wnt pathway by transfecting Dkk-3 and dominant-negative LRP5 into osteosarcoma cells significantly reduced invasion capacity and cell motility potentially by recruiting *β*-catenin to the cell surface to promote cell-cell adhesion [20]. This is supported by other studies in which the Wnt pathway was downregulated by genetic or drug-treatment approaches in osteosarcoma cells, and as a result the decreased invasion and motility were observed, with the related clinical observations being an antitumor and antimetastasis effect [18, 29, 31, 32]. Furthermore, the activation of Wnt pathway and increased nuclear *β*-catenin localization has been detected in the majority of osteosarcomas and may correlate with metastasis [14, 33]. This is recapitulated in canine osteosarcoma which is very similar to the human disease [34]. Interestingly, on the other hand, a group has performed expression profiling of high-grade osteosarcoma, MSCs, and the same MSCs differentiated into osteoblasts, and osteoblastomas and has suggested that downregulation of Wnt signaling has an important role in osteosarcoma pathogenesis [35]. That same group has also reported the loss of Wnt/*β*-catenin pathway activity in osteosarcoma cells by measuring the nuclear *β*-catenin level, and using a GSK3*β* inhibitor [36]. Although the authors discussed these results as being contradictory with other reported studies, factors involved in pathogenesis and progression are frequently not the same. In this study, after transfecting *β*-catenin into MSC-TS, clones with different *β*-catenin level were deliberately picked to detect the effects of *β*-catenin on pathogenesis. No significant changes were detected among clones both phenotypically and functionally. Moreover, no tumor formation was observed in mice. These findings support the view that the expression of *β*-catenin alone is not involved in osteosarcoma pathogenesis regardless of the activity of the Wnt-signaling pathway. Frequent *β*-catenin accumulation in osteosarcoma may relate to the stage at which most cases become clinically apparent rather than reflecting events early in pathogenesis [14]. The other possibility could be generating a model with active canonical Wnt signaling, for example, transfection with constitutively active LRP5/6. An additional explanation which needs to be considered is the tested genetic manipulations and tumor-specific alterations which have utilized and tested different components of this complicated signaling pathway, which complicates the interpretation of results. As an example, manipulation of Dkk-3 may be either context-dependent or may potentially produce some affects independent of *β*-catenin or other selected Wnt pathway components [17, 32].

A potentially important observation is that after *β*-catenin transfection into MSC-TS cell lines, the full multilineage differentiation capacity of the parental MSCs was lost. When MSC-TSBs were induced to osteoblasts, adipocytes, and chondrocytes via the respective induction media, osteogenesis was favored. No adipogenic differentiation was observed and chondrogenesis was delayed. This finding is also supported by previous studies in which inactivating mutations in LRP-5, Wnt coreceptor, are associated with decreased bone mass, as a consequence of reduced osteoblast differentiation and vice versa [37–40]. Loss of adipogenicity of MSC-TSB cells upon induction is worth noting here because osteosarcoma does not have an adipocytic subtype variant, while osteoblastic, chondroblastic, and fibroblastic osteosarcoma subtypes do exit [3, 4]. Osteosarcoma may therefore derive from such an intermediate stage of differentiated MSCs. Current descriptions of mesenchymal stem cell do not describe intermediate stages of differentiation in which cells have become committed to a restricted set of final lineages. Comparable to hematopoiesis, it is quite likely that intermediate stages of differentiation exist. These results suggest that adipogenesis may be an early branch point in mesenchymal differentiation. It is possible in normal mesenchymal stem cell differentiation that a stem cell which has the capacity to differentiate into chondrogenic and osteogenic but not adipogenic lineages may exist. A caveat of this suggestion is that these results were obtained with transformed cells and may not reflect what occurs with normal mesenchymal stem cell differentiation.

In conclusion, this study demonstrated the addition of *β*-catenin to MSC-TS cells did not result in cells with tumorigenic properties. This suggests that *β*-catenin may not have a role in osteosarcoma pathogenesis; however, this does not rule out its potential involvement in osteosarcoma progression. The generation of cells with the capacity to differentiate into selected but not all mesenchymal stem cell lineages suggests that intermediate stages may exist. Further studies will be necessary to clarify the role of other components of the Wnt-signaling pathway in osteosarcoma pathogenesis and progression.

## Abbreviations

MSC: Mesenchymal stem cells
hMSC: Human mesenchymal stem cells
MSC-TS: Human mesenchymal stem cells transfected with hTERT and SV40 large T antigen
MSC-TSB: MSC-TS transfected with *β*-catenin
MSC-TSR6: An MSC-TS subline transfected with activated H-Ras
ECM: Extracellular matrix.
